# Severe Cardiac Toxicity Induced by Cancer Therapies Requiring Intensive Care Unit Admission

**DOI:** 10.3389/fcvm.2021.713694

**Published:** 2021-09-03

**Authors:** Andrea Montisci, Vittorio Palmieri, Jennifer E. Liu, Maria T. Vietri, Silvia Cirri, Francesco Donatelli, Claudio Napoli

**Affiliations:** ^1^Division of Cardiothoracic Intensive Care, Azienda Socio-Sanitaria Territoriale (ASST) Spedali Civili, Brescia, Italy; ^2^Department of Cardiac Surgery and Transplantation, Ospedali dei Colli Monaldi-Cotugno-CTO, Naples, Italy; ^3^Department of Medicine/Cardiology Service, Memorial Sloan Kettering Cancer Center, New York, NY, United States; ^4^Department of Precision Medicine, University of Campania “Luigi Vanvitelli”, Naples, Italy; ^5^Department of Anesthesia and Intensive Care, Istituto Clinico Sant'Ambrogio, Milan, Italy; ^6^Department of Cardiac Surgery, University of Milan, Milan, Italy; ^7^Clinical Department of Internal Medicine and Specialistics, University Department of Advanced Clinical and Surgical Sciences, University of Campania “Luigi Vanvitelli”, Naples, Italy; ^8^Istituto di Ricovero e Cura a Carattere Scientifico - Synlab Diagnostica Nucleare (IRCCS SDN), Naples, Italy

**Keywords:** anthracycline, mechanical circulatory support, heart failure, heart transplant, cancer, chemotherapy, chemotherapy toxicity

## Abstract

A steadying increase of cancer survivors has been observed as a consequence of more effective therapies. However, chemotherapy regimens are often associated with significant toxicity, and cardiac damage emerges as a prominent clinical issue. Many mechanisms sustain chemotherapy-induced cardiac toxicity: direct myocyte damage, arrhythmia induction, coronary vasospasm, and accelerated atherosclerosis. Anthracyclines are the most studied cardiotoxic drugs and represent a clinical model for cardiac damage induced by chemotherapy. In patients suffering from advanced heart failure (HF) because of chemotherapy-related cardiomyopathy, when refractory to optimal medical therapy, mechanical circulatory support or heart transplantation represents an effective treatment. Here, the main mechanisms of cardiac toxicity induced by cancer therapies are analyzed, with a focus on patients requiring intensive care unit (ICU) admission during the course of the disease because of acute cardiac toxicity, takotsubo syndrome, and acute-on-chronic HF in patients suffering from chemotherapy-induced cardiomyopathy. In a subset of patients, cardiac toxicity can be acute and life-threatening, leading to overt cardiogenic shock. The management of critically ill cancer patients poses a unique challenge and requires a multidisciplinary approach. Moreover, no etiologic therapy is available, and only supportive measures can be implemented.

## Graphical Abstract

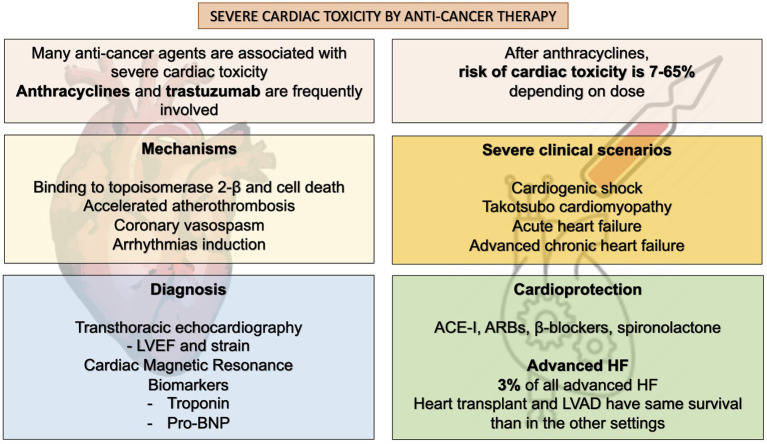

## Introduction

In the last decade, we observed a continuous increase of cancer incidence, accompanied a by stable decrease of cancer-related mortality. Early diagnosis and effective treatments mainly account for this epidemiological and clinical shift, which led to an increase of cancer survivors (1).

The efficacy of chemotherapy regimens and new biological and immunological treatments is often associated with a significant cardiovascular toxicity.

The patients at high risk for severe toxicity development are those with preexistent cardiovascular diseases or with multiple risk factors, associated with high dose of cardiotoxic drugs, mainly anthracyclines (2).

Moreover, the development of cardiotoxicity often causes the interruption of an effective treatment, preventing the completion of the therapy and influencing the oncologic prognosis.

Cardiac damage requiring intensive care unit (ICU) admission and advanced heart failure (HF) therapies for chemotherapy-induced cardiomyopathy (CCMP) will be also investigated.

## Cancer Therapy-Induced Cardiac Damage

### Pathogenic Mechanisms

Cancer therapy may induce myocardial damage by various mechanisms, as each anticancer agent has unique cardiac effects such as direct myocyte toxicity leading to HF or inhibition of ion channels or tyrosine kinase signaling pathways leading to arrhythmias with or without QT prolongation.

Besides, anticancer therapy may trigger coronary atherothrombosis, pulmonary thromboembolisms, and arterial hypertension (HTN), determinants of myocardial damage and cardiac remodeling.

The association of cardiovascular risk factors with the use of chemotherapy represents a major issue in terms of increased risk of myocardial damage, with or without a specific histological pattern.

#### Anthracyclines

Anthracyclines are the best studied among the anticancer drugs with established cardiotoxicity. Discovered in the 1960s, they remain as some of the most effective anticancer drugs mostly used to treat solid and hematologic cancers (3), such as lymphoma, leukemia, breast cancer, and sarcoma. Anthracyclines may induce cardiac toxicity acutely or at distance from single or complete treatments. In particular, doxorubicin and epirubicin are used in breast cancer, before or following surgery, and in patients with metastatic diffusion. While oxidative stress, DNA damage and impairment of DNA repair through inhibition of the topoisomerase II, activation of senescence, and cell death are all commonly reported mechanisms of anthracycline-induced toxicity (4), the reasons for a specific myocardial, cumulative-dose depending damage remains largely unclear. The binding to topoisomerase type 2 beta, largely expressed in cardiac myocytes, is thought as one of the main mechanisms for the specific cardiac toxicity of anthracyclines resulting in cell death (5).

Longitudinal studies of childhood cancer survivors showed that even children who received <250 mg/m^2^ had an increased risk of congestive HF on long-term follow-up, suggesting that there is no safe threshold for anthracycline-induced cardiotoxicity. With the use of multi-agent combination therapy for definitive cancer cure, cardiotoxic effects have been observed without a specific threshold of cumulative dose, especially if treatment is combined with radiotherapy, cyclophosphamide (6), paclitaxel, and trastuzumab (7) and if cardiovascular risk factors such as HTN and diabetes are present (8). There is active investigation in identifying pharmacogenomic predictors of anthracycline-induced cardiotoxicity, as individual susceptibility is variable. Clinical evidence of anthracycline-induced cardiotoxicity most often occurs within the first year of treatment, although it can also appear years later with signs and symptoms of HF. Acute cardiotoxicity is rare. Cardiac phenotype associated with anthracycline-induced cardiomyopathy includes dilated left ventricle (LV) with myocardial dysfunction, dilated atria, mitral and tricuspid insufficiency secondary to ventricular enlargement and dysfunction, increased filling pressure, and reduced cardiac output.

#### Fluoropyrimidine

The main mechanism of action of fluoropyrimidine drugs including 5-fluorouracil (5-FU) and capecitabine is inhibition of pyrimidine nucleotide biosynthesis and interference with DNA synthesis and mRNA translation. The toxicity associated with fluoropyrimidine chemotherapy affects almost 30% of patients (9, 10). The most known cause of fluoropyrimidine toxicity is the deficiency of dihydropyrimidine dehydrogenase (DPD), a crucial enzyme in fluorouracil metabolism, which is encoded by *DPYD* gene (11). The most common symptom of fluoropyrimidine cardiotoxicity is chest pain, which can be associated with other symptoms such as palpitations, dyspnea, HTN, or hypotension. Less common manifestations include myocardial infarction, reversible cardiomyopathy, myopericarditis, congestive HF, tachyarrhythmias, coronary dissection, and cardiogenic shock (CS) (12). Recently, cardiotoxicity associated with 5-FU has been reviewed in detail (13). From a clinical point of view, the 5-FU may be associated with atypical chest pain, effort chest pain (14), or suspected acute coronary syndrome (15) with or without ST segment elevation. In addition, chest pain and atrial fibrillation may be associated with 5-FU treatment and release of cardiac specific enzymes, in the absence of ST segment deviation, suggesting myocarditis and pericarditis (16) up to and rapidly progressive LV dysfunction and HF (17). Nevertheless, relatively sudden and rapidly progressive events resolving in death occur in <1% (18). *In vitro* studies using rabbit aorta rings (19) or studies in brachial artery (20) or coronary arteries with ECG evaluation following 5-FU administration (21, 22) suggested vasospastic reaction of relatively small muscle arteries. Nevertheless, the mechanism is not specific and consistent and is mostly related to underlying atherosclerosis (23). In a study, evidence of global LV wall motion abnormalities has been reported during chest pain and ECG changes suggestive of myocardial ischemia following 5-FU administration (15), which is unlikely to be dependent on coronary vasospasm, also because it may occur hours or days after the infusion. Mechanisms involving endothelial-dependent and endothelial-independent dysfunction related to 5-FU administration may affect coronary microvasculature and may be described in the absence of epicardial vessels abnormalities (24). It should be taken into account that endothelial function and microvasculature autoregulation influence, and is also influenced by, platelet aggregation and pro-thrombotic factors, which may be altered with 5-FU administration (25). Endothelial cells accelerated turnover described with 5-FU administration has been demonstrated using von Willebrand factor dosing as a potential result of toxic free radicals released (26).

#### Alkylating Agents

Anticancer agents such as cyclophosphamide, ifosfamide, and melphalan are defined as alkylating agents. Those agents ultimately impact DNA transcription and protein synthesis (27) and may induce cardiac damage early after administration, with a relatively strong predicting factor represented by the cumulative dose (cyclophosphamide, 150 mg/kg, 1.5 g/m^2^ per day; ifosfamide, ≥12.5 g/m^2^). Of note, the prevalence of subjects presenting with symptomatic or pauci-symptomatic ventricular systolic dysfunction secondary to use of alkylating agents is comprised between one and three per 10 patients treated, approximately.

#### Microtubular Polymerization Inhibitors

The so-called taxanes are anticancer chemotherapeutic agents including paclitaxel and docetaxel, able to block cell division through a mechanism of binding and inhibition of disassembly of microtubules (28). Cardiac dysfunction, either asymptomatic or symptomatic, is unlikely with those agents. However, taxanes impact metabolism and excretion rate of anthracyclines and therefore may increase the risk of anthracycline-related development of HF (29, 30), which may be attenuated by specific modifications of treatment protocols. Novel epigenetic mechanisms and epidrugs are now involved in the natural history of HF and its treatment (31).

#### Anti-HER2 Therapy

##### Cancer Chemotherapy by Monoclonal Antibodies Against the Human Epidermal Growth Factor Receptor 2 HER2/ERbB2

Trastuzumab is a humanized monoclonal antibody against the extracellular domain of an oncoprotein identified as human epidermal growth factor receptor 2 (HER2/ERbB2), which is highly relevant in prognosis in breast cancer (32–35).

Moreover, the use of trastuzumab increased the incidence of cardiac toxicity due to use of anthracyclines. The reason for such a negative interaction may lay in the fact that HER2/ERbB2 exerts a protective role against myocardial damage (36), with prevalent ventricular dysfunction in post-chemotherapy as high as three in 10 patients treated (37).

The introduction of new agents, such as lapatinib and pertuzumab, new monoclonal antibodies against different targets of HER2, has reduced the likelihood of developing permanent cardiac damage secondary to toxicity, as they are frequently used in combination with trastuzumab and docetaxel with new protocols of treatment (38, 39). There are now available combinations of HER2-targeted antibody with a cytotoxic agent such as anthracyclines, arising new questions on incident cardiotoxicity in treated patients (40).

#### Vascular Endothelial Growth Factor Inhibitors (Monoclonal Antibodies and Small Molecules)

By a variety of mechanisms (41), vascular endothelial growth factor (VEGF) inhibitors inhibit angiogenesis. There are small molecules (sunitinib and sorafenib) able to determine non-selective inhibition of VEGF receptors through inhibiting different tyrosine kinases. The non-selective inhibition of VEGF may be the reason for development of HTN as well as atherosclerosis and atherothrombosis in patients being treated (42–44). HTN is the most common side effect (45). The exact mechanism of angiogenesis inhibitor-induced HTN is not completely understood; several hypotheses have been proposed. Nitric oxide (NO) has been shown to affect vascular smooth muscle relaxation; there is evidence that VEGF signaling can affect NO production and homeostasis. VEGF binding with the VEGFR2 activates several intracellular signaling pathways that upregulate the expression of endothelial NO synthase that leads to vasodilation. VEGF inhibition decreases the production of NO leading to vasoconstriction, peripheral vascular resistance, and HTN (46). Additionally, impaired NO production affects renal sodium homeostasis, leading to further elevations in blood pressure (47). Rarefaction is another postulated mechanism through which VEGF inhibition can lead to HTN. This process involves a decrease in capillary density at the peripheral level with increased vascular resistance. This phenomenon is thought to be reversible after discontinuation of the VEGF inhibitor (48). VEGF inhibition may also lead to increased production of other vasoactive substances, for example, sFlt-1 and endothelin-1 (ET1), which contribute to the development of HTN (49).

The anti-VEGF antibody bevacizumab has been tested in innumerous trials in which HTN, bleeding, and thrombosis emerged as the main cardiovascular adverse effects (50).

Post-marketing surveillance suggests that more than one patient in 10 treated with those agents may have experienced symptomatic reduction of the LV chamber function (42), with a risk particularly elevated in those treated with bevacizumab (51).

### Clinical, Imaging, and Laboratory Characteristics

#### Cardiovascular Imaging and Allied Diagnostic Technology

Cardiac ultrasound is the most commonly used method for assessing cardiac structure and function in the ambulatory setting as well as bedside and in urgency/emergency setting.

In the setting of emergency medicine, ultrasounds are employed to assess lung structure, pleural effusion, and abdominal and vascular areas contributing to differential diagnosis, risk stratification, and response to treatments (52).

In subjects exposed to anticancer chemotherapy, echocardiography is the recommended modality (53) for assessment of ventricular chamber dimensions and shortening, diastolic performance, valvular function, and right ventricular (RV) load by assessing the inferior vena cava diameter and peak velocity of the tricuspid insufficiency to derive peak systolic pulmonary pressure, atrial dimensions, and pericardium and pulmonary characteristics, which can be also extrapolated by ultrasound imaging. LV ejection fraction (LVEF) is a parameter of LV systolic function influenced by load and geometry (54, 55).

As global LVEF may be informative, segmental systolic abnormalities in the context of substantially normal LVEF may be relevant in patients with cancer, impacting treatment dose adjustment or even decision to suspend treatments (56). New ultrasound technology allows assessment of LVEF by a real-time three-dimensional imaging modality, useful in persons exposed to anticancer chemotherapy with improved accuracy as compared with standard imaging modality (57). However, real-time three-dimensional echocardiography is not used in routine practice, yet.

In the last decade or two, new methods of quantification of the myocardial systolic deformation have been developed and tested also in patients with exposure to anticancer chemotherapy (58). The so-called 2D speckle-tracking modality allows assessment of ventricular and atrial wall systolic and diastolic deformations and deformation rate, defined as strain and strain rate. Those parameters are thought to be less load and operator dependent. It has been reported that peak systolic global longitudinal strain of the LV may be impaired, while LVEF may be normal in patients who may develop HF later in follow-up (59). In addition, peak systolic longitudinal global strain may be impaired despite that LVEF may be found at least partially recovered in patients who received high-dose anthracyclines (60).

Peak systolic longitudinal global strain was able to discriminate among patients who received trastuzumab alone or with anthracyclines and found that those who showed a difference between follow-ups of >11% had the highest likelihood to show cardiotoxicity (61). Assessment of the peak systolic global longitudinal strain is recommended for LV systolic function follow-up in subjects exposed to anthracyclines (62). However, such a modality for quantification of chamber function remains most as a research tool in the real-life world.

Ultrasound imaging can be used to explore also the diastolic phase of the cardiac mechanic in patients exposed to anticancer chemotherapy (63), with inconsistent albeit encouraging data (64).

Still, routine assessment of diastolic parameters to predict subsequent cancer-related cardiotoxicity remains more a research-oriented resource (65).

Anticancer chemotherapy has been associated with right myocardial damage detected by biopsy (66).

RV chamber size, tricuspid annular plane systolic excursion, estimation of pulmonary artery systolic pressure, and RV diastolic parameters are useful parameters in the follow-up of subjects who underwent anticancer chemotherapy (67), along with additional laboratory parameters related to cardiovascular overload and HF, such as N-terminal pro-brain natriuretic peptide (NP) (NT-proBNP) levels (68).

In patients with symptoms and signs suspected of HF and/or myocardial ischemia, global LVEF may be relatively preserved despite increased LV filling pressure (69). The pulmonary systolic pressure (67) and the ratio of peak velocity of the early LV filling wave to the peak velocity of the early diastolic displacement of the mitral annulus >13 may contribute to identifying patients with acute HF (70) independent of LVEF. Along with echocardiographic examinations and lung, vascular, and abdominal ultrasound evaluations (71) for assessing pulmonary interstitial syndromes (B-lines), pleural effusion and lung consolidation vs. lung atelectasis, central venous dimensions and reactivity to maneuvers, liver congestion, and ascites are very useful for triaging patients, for diagnosis, and to monitor response to treatments.

In more stable patients, either ambulatory or hospitalized, cardiac structure and functions may be explored by cardiac magnetic resonance imaging (cMRI). In particular, cMRI may be useful to detect cardiac damage and its temporal relationship of myocardial damage induced by anticancer chemotherapy (71, 72). In fact, myocardial edema, inflammatory injury, and fibrosis may be detected by cMRI (73, 74), as it is largely employed in models of myocardial injury based on inflammation. Assessment of myocardial edema and vascular damage may be prognostically relevant well beyond the extent of late gadolinium-related myocardial enhancement in patients exposed to anticancer chemotherapy (75, 76).

Allied imaging modality includes cardiac computed tomography (CT) to assess coronary artery calcium burden scanning and CT coronary angiography. In particular, non-contrast-enhanced CT to evaluate coronary artery calcium burden (77) reflects coronary atherosclerotic burden with important prognostic value for future adverse cardiac events in asymptomatic individuals (78), which may turn relevant in candidates for or treated with anticancer chemotherapy.

Positron emission tomography (PET), which is widely used to evaluated cancer diffusion and response to treatments, may be useful in evaluating inflammatory-induced myocardial injury (79). Cardiac PET has been shown to be useful in the model of non-Hodgkin lymphoma receiving chimeric antigen receptor T-cell transfer therapy correlated with the degree of cytokine release syndrome, in myocarditis, pericardial effusions, and response to treatment in a patient affected by large B-cell lymphoma with cardiac involvement (80).

#### Laboratory Characteristics

Traditionally, subclinical cardiac toxicity has been detected by evaluating the reduction of LVEF with the use of echocardiography or other imaging. Although useful, this strategy is limited by two factors: there is significant myocardial damage when the decline in LVEF is still not evident, and imaging takes time and is impractical when used as a screening modality.

Biomarkers may help to identify subjects who may develop or have developed chemotherapy-related cardiotoxicity; therefore, for an early diagnosis of myocardial toxicity, the dosing of cardiac biomarkers becomes an alternative strategy (81). The time of cardiac biomarker measurement in cancer patients scientifically has not been established. The timing and frequency of biomarker measurement should be tailored to each biomarker–therapy combination. This could be translated into earlier detection and implementation of cardioprotective treatment strategies in cancer patients (82).

Cardiac troponin (cTn) and both troponin I (TnI) and troponin T (TnT) are the gold standard biomarkers for the detection of cardiomyocyte necrosis and cardiac injury and the most extensively used biomarker to detect cardiac toxicity (83). Both increase 4–6 h after the onset of symptoms, peak after 14–36 h, and return to normal levels after 7–8 days (cTnI) or after 12 days (cTnT) in acute coronary syndromes and myocardial infarction. They have high specificity for cardiac injury; moreover, with the advent of high-sensitivity (hs) assays, it is possible to detect small amounts of myocyte damage to provide treatments to minimize cardiotoxicity before the development of irreversible LV dysfunction (84). The importance of monitoring troponin to detect cardiotoxicity has been demonstrated from studies of cancer patients receiving chemotherapy, mainly anthracyclines (85). A persistent elevation of troponin I was associated with a higher incidence of cardiac events and a greater degree of LV dysfunction as compared with transient elevations (84, 86). The optimal timing in monitoring cardiotoxicity for cTn measurement has not yet been determined; an improved understanding of the kinetics of cTn release and optimal timing for blood sampling during anthracycline chemotherapy would be useful for anthracycline cardiotoxicity surveillance (87).

Clinical studies in both children and adults have shown that elevated troponin levels during chemotherapy are an early marker of increased risk of LV dysfunction (88–90).

The largest study to date involved 703 oncologic patients where troponin I was measured before chemotherapy, within 3 days of chemotherapy start and after 1 month (84).

Troponin I was in the normal range in 70%, increased at 3 days in 21% of patients, and increased in both early and late stages in 9% of patients. The elevation of troponin I was associated with a higher incidence of adverse cardiovascular events; troponin I levels that still increased after 1 month were associated with greater LV impairment and a higher incidence of cardiovascular events as compared with an isolated elevation at 3 days (84). Another study involving 204 patients with cancer requiring anthracyclines involved measuring troponin for every cycle of high-dose chemotherapy (91); in 32% of patients, elevated troponin is observed. Both studies, hence, validate the importance of using troponin for surveillance post-chemotherapy.

The dosage, therefore, of the TnI can allow the initiation of cardioprotective therapy and more specific treatments to prevent clinically significant toxicities (92).

Other increasing data emerging from patients with childhood cancers show that cTnI and hs-TnI were not consistently elevated in patients who developed LV dysfunction on imaging (93, 94). From these data, it emerges that although troponin is a sensitive marker during and early after chemotherapy, it could not be a sensitive marker for ongoing surveillance of subclinical cardiotoxicity post-chemotherapy, when compared with other biomarkers such as NPs.

Few studies prospectively investigated the changes in TnI or TnT levels during 5-FU chemotherapy. In three studies, no significant increase in TnI was noted after 5-FU infusion, although a group of patients developed cardiac symptoms or ECG abnormalities (95–97). In a study by Salepci et al., TnT levels were assessed in five samples during the 5-FU bolus cycle; no changes were observed before and immediately after chemotherapy; however, three patients developed chest pain, five patients had ECG changes suggestive of ischemia, and one patient died (98).

In another study, Holubec et al. measured both TnI and BNP before and after infusion of 5-FU, highlighting a rise in TnI levels above the normality range in 57% of patients (99).

The European Society for Medical Oncology (ESMO), in their clinical practice guidelines regarding fluoropyrimidine cardiotoxicity, recommends monitoring of TnI and BNP in patients with symptoms or signs of cardiac ischemia as a grade C III/IV level of evidence (100).

Changes in troponin levels as a strategy to monitor patients receiving anti-VEGF monoclonal antibodies, anti-VEGFR tyrosine kinase inhibitors, and a kinase inhibitor have been used with some promising results, although more useful to research context than clinical setting (101).

In patients suffering from immune checkpoint inhibitor (ICI)-related myocarditis, an increase of TnT ≥ 1.5 ng/ml was associated with four-fold increased risk of major adverse cardiac events (MACEs), defined as the composite of cardiovascular death, CS, cardiac arrest, and hemodynamically significant complete heart block (76).

Another study reports two melanoma patients who developed fatal myocarditis following treatment with ipilimumab and nivolumab. ECG and weekly troponin levels during weeks 1–3 for patients on combination immunotherapy were evaluated. Both patients experienced strikingly elevated troponin levels and refractory conduction system abnormalities with preserved cardiac function (102).

Moreover, in a study, 65% of advanced renal cell carcinoma patients treated with sunitinib developed a form of cardiovascular toxicity. The cardiac troponins were not found much useful to describe this cardiotoxicity, due to the low incidence of troponin elevation in patients uncovered by this analysis. This could mean that sunitinib-induced cardiotoxicity is different from that induced by other chemotherapeutic drugs and requires different markers for its detection (51).

Two NPs, i.e., BNP and NT-proBNP (103), are stimulated to be secreted by cardiomyocytes from increased transmural tension and neurohormonal stimulation. BNP is derived from cleavage of pre-proBNP leading to BNP and the biologically inactive N-terminal-containing fragment (NT-proBNP). NT-proBNP has also been used as a biomarker for the detection of HF (104).

Hence, NPs are an important biomarker of pressure overload, and their ability to detect hemodynamic stress makes them an important maker of cardiotoxicity and for long-term surveillance in the management of HF. NPs may also be used to detect acute cardiotoxicity as levels increase within 24 h of exposure to anthracycline chemotherapy (105). Also, BNPs can be considered as a marker of cardiotoxicity. A single-center study of 109 cancer patients undergoing treatment with anthracyclines showed that 10.1% experienced a cardiac event, and all of these patients had a BNP > 100 ng/L before the event (105). This supports the regular BNP measurements as well as imaging to detect cardiotoxicity. In the HERA breast cancer trial of trastuzumab, the cardiac biomarker substudy reported that increases in NT-proBNP during trastuzumab treatment were most predictive of a subsequent reduction in LV function on echocardiographic surveillance (106). In another study that involved 43 patients with breast cancer after radiotherapy, serial BNP measurements up to 12 months' post-therapy have been performed; it was observed that small BNP elevations were predictive for the development of radiotherapy-related cardiovascular events, but none of the patients developed LV dysfunction (107).

Two prospective studies evaluated changes in BNP levels by performing serial measurement in patients treated with infusional 5-FU. This study showed an increase in BNP above the normal values in patients treated with infusional 5-FU; this increase was significantly higher in patients who were symptomatic for cardiotoxicity than in asymptomatic patients, but these data were not able to indicate a cutoff to distinguish patients with cardiotoxicity, and the role of BNP as a predictor of fluoropyrimidine-induced cardiotoxicity (FIC) remains to be clarified (108).

Troponin is a biomarker that defines cardiomyocyte injury, whereas BNP is a marker of increased myocardial strain. NPs are excellent markers of long-term cardiovascular dysfunction in asymptomatic patients. Other biomarkers provide information on the acute injury but may not remain elevated in the long term (92).

C-reactive protein (CRP) is an acute-phase reactant produced by hepatocytes due to stimulation by interleukin-6 produced by macrophages and T cells. Elevated hs-CRP is associated with decreased LVEF in patients with varying cardiovascular diseases (109).

A study involving 54 women treated with trastuzumab showed that peak levels of hs-CRP were detected after a median of 78 days before cardiotoxicity became evident as quantified by a decrease in LVEF. However, other studies failed to show a conclusive link between elevated CRP and cardiotoxicity (110, 111).

The limitation of hs-CRP is that is has a low specificity when elevated to 45.7%; hence, due to the risks of achieving false-positive values, a high level does not confirm the development of LV dysfunction (112).

The modifications during chemotherapy with 5-FU of the cardiac enzymes creatine kinase (CK) and CK-MB have been analyzed in three different studies, but no significant differences before and after 5-FU infusion were detected (113).

Myeloperoxidase (MPO) (produced and secreted by leukocytes) is an enzyme produced by polymorphonuclear leukocytes and has atherogenic and pro-oxidant effects on cardiac tissue leading to its association with increased risk of coronary artery disease and acute HF (114).

MPO has been shown to work synergistically with troponin to predict adverse cardiovascular outcomes.

The first description of the link between an increase in MPO levels and the development of cardiotoxicity was described following treatment with doxorubicin and trastuzumab (90), in which the clinical significance of MPO elevation has been highlighted.

In conclusion, the integrated use of imaging and biomarkers can help determine the basic risk of cardiotoxicity and identify patients who may benefit from cardiac monitoring or cardioprotective pharmacological strategies.

### Clinical and Therapeutic Perspectives in Intensive Care Unit

In the past, due to the disappointing results of therapies in solid tumors, ICU admission for cancer patients was frequently denied. In the last decades, however, the progress of cancer therapy, which allowed for a significant improvement in patients' prognosis, opened the doors of ICU to cancer patients. Patients suffering from hematologic malignancies and solid tumors need ICU admission not only because of the complications of the disease or the surgery but, significantly, because of the toxic effects of cancer therapies. Cardiac toxicity is of paramount importance, as it can virtually accompany patients who received cardiotoxic cancer therapy throughout their lives, starting from the risk of acute toxicity at the time of first administrations up to the late effects of advanced HF.

Moreover, the number of discovered drugs that have been proved cardiotoxic is increasing, including newer immunotherapeutic agents.

#### Intensive Care Unit and the Cancer Patient

Many cancer patients are admitted to ICU during the course of their disease. Admission rate for a non-selected population of cancer patients in France ranged from 0.7 to 12%, with patients suffering from hematologic malignancies showing an ICU resource utilization of up to 30% (115).

ICU and hospital survival of critically ill cancer patients also improved, with a mortality rate below 30 and 40%, respectively (90). Usual triage criteria for ICU admission are often unreliable, and new models of ICU admission policies have been developed (116).

The most frequent causes of ICU admission are infections, tumor lysis syndrome, acute kidney injury, acute respiratory failure, regular postsurgical care, cytokine release syndrome, neurological complications and adverse drug effects, chemotherapy-induced severe neutropenia, and cardiac toxicity (117, 118).

#### Cardiac Toxicity Requiring Intensive Care Unit Admission

General criteria for ICU admission do not differ from usual indications: the development of CS, hemodynamic instability, need for invasive mechanical ventilation or organ function replacement therapy, extracorporeal therapies, and “prophylactic” ICU admission in patients deemed at high risk to develop such complications in a short timeframe.

CS is the most dreadful condition, associated with a high mortality rate. To the best of our knowledge, there are no studies directly focused on CS in patients with severe cancer-related diseases. A large European registry (119) reported a 4.3% prevalence of cancer in patients with CS, without further information on causes. CS can be the consequence of acute cancer therapy toxicity, takotsubo cardiomyopathy (TCM), or acute-on-chronic HF in patients suffering from CCMP.

The therapy of CS in this subset of patients lacks a specific therapy, and only supportive measures are available. These include mechanical ventilation, inotropes and mechanical circulatory support (MCS), intra-aortic balloon pump, percutaneous ventricular assist devices, and veno-arterial extracorporeal membrane oxygenation (VA ECMO) (120).

A directed therapy is only available for CS caused by acute toxicity after 5-FU/capecitabine administration or overdose, burdened by a very high mortality rate if left untreated.

The antidote uridine triacetate protects against the toxic effect of fluoropyrimidines and can be safely administered. Ma et al. (121) reported a 94% survival in 173 patients (26 had early onset of toxicity, whereas 147 suffered from overdose) treated with uridine triacetate. In patients with early toxicity, a more favorable outcome was associated with a therapy initiation before 96 h from the onset.

Cancer patients requiring ICU admission for cardiac toxicity can be classified into three main categories: acute cardiac toxicity caused by anthracyclines or 5-FU administration; takotsubo syndrome; and acute-on-chronic HF in patients suffering from CCMP.

##### Acute and Early Cardiac Toxicity

Anthracyclines and 5-FU are the most frequently involved drugs.

According to an old classification, dating back to 1980, acute toxicity is defined as the onset of LV dysfunction or arrhythmias during or within 14 days from the end of treatments (122). Clinical characteristics include transient arrhythmias as supraventricular tachycardia, non-specific ST segment or T wave abnormality, pericardial myocarditis syndrome, or acute LV failure. Ventricular arrhythmias have been seldom described (123).

In childhood cancer patients, it occurs in <1% of patients; cardiac dysfunction is usually transient, and cancer therapy can be resumed. In patients with a high cumulative dose of anthracyclines, the acute damage can be permanent (124).

Acute toxicity has never been the object of large clinical studies, and the description of clinical characteristics mostly relies on case reports. However, the first dose of anthracycline, whether or not associated with clinical expression of cardiac damage, is accompanied by a release of troponin in nearly all treated patients, reinforcing the concept of a continuum of toxicity, evolving toward overt HF through years or decades (125).

5-FU and capecitabine are fluoropyrimidines employed in a variety of solid tumor therapy, frequently involved in episodes of acute toxicity. In the majority of cases, cardiac toxicity is self-limiting and manifests as chest pain with or without ST segment depression.

5-FU- and capecitabine-related early toxicity usually manifests during or within the 96 h after the administration of 5-FU or during a standard 14-day course of capecitabine (days 3–9).

A systematic review (126) on cardiotoxicity in cancer patients treated with 5-FU or capecitabine showed an incidence of 0–18.6%. Serious cardiac events as myocardial infarction, CS, and cardiac arrest occurred in 0–2%. These events have usually a high fatality rate.

A study reported a 4.3% incidence of cardiac toxicity in 668 patients treated with 5-FU or capecitabine for gastrointestinal cancers (127). Severe cardiac toxicity is rare and reported in case reports only: coronary dissection, ventricular tachyarrhythmia, CS (requiring intra-aortic balloon pump and VA ECMO), and sudden cardiac death.

Interestingly, after a first episode of cardiac toxicity, readministration of 5-FU can induce a more severe form of cardiac damage, with a mortality rate up to 13% (126, 127).

Treatments that specifically target the HER2 receptor, as trastuzumab and pertuzumab, can be responsible for acute cardiac toxicity, usually reversible (128). Few cases needing intensive care treatment have been described (129).

##### Myocarditis

Myocarditis is an uncommon, but potentially fatal, toxicity of ICIs.

In a study (76), 35 patients with ICI-associated myocarditis were compared with a random sample of 105 ICI-treated patients without myocarditis. The occurrence of MACEs, defined as the composite of cardiovascular death, CS, cardiac arrest, and hemodynamically significant complete heart block, was evaluated in this multicenter cohort. The prevalence of myocarditis was 1.14%, with a median time onset of 34 days from the first infusion. Combination ICI therapy and diabetes were more common in some cases; 46% of all myocarditis cases experienced a MACE. Myocarditis often showed a fulminant and malignant course. Causes of death included two sudden deaths, two documented ventricular arrhythmias, and two CSs.

High-dose intravenous steroids were the most commonly administered therapy.

Escudier et al. (130) reported 30 cases of ICI-related cardiac toxicity, with an onset 2–454 days after the first dose (median 65 days). LV systolic dysfunction, takotsubo-like syndrome, atrial fibrillation, ventricular arrhythmias, and conduction disorders were observed in 79, 14, 30, 27, and 17% of patients, respectively. Cardiovascular mortality rate was 27%, due to refractory ventricular arrhythmias, HF, pulmonary embolism, and sudden death.

Interestingly, complete reversibility of LV systolic dysfunction was significantly associated with corticosteroid therapy.

The fulminant course of the ICI-related myocarditis has been confirmed by Moslehi et al., who reported a 46% mortality rate in 101 cases. In this cohort, median onset from the first dose was 27 days (131).

As regard treatment, no prospective studies have been conducted. The treatment with ICI must be promptly suspended. On the basis of the available case series, Gunatra et al. suggested high dose of corticosteroids (i.e., methylprednisolone 1,000 mg per day for 3 days followed by prednisone 1 mg/kg) as the first line of therapy in the acute phase. If patient is unstable, anti-thymocyte globulin, intravenous immunoglobulin, and plasma exchange should be considered (132).

Potential alternatives to steroids in the American Society of Clinical Oncology (ASCO) guidelines include methotrexate, mycophenolate mofetil, azathioprine, and rituximab. Infliximab is contraindicated due to its potential to induce HF (133).

A better understanding of the mechanism of this drug-induced toxicity may provide valuable insight into idiopathic myocarditis in the non-cancer population, as well as the general interaction between the immune system with the myocardium (102).

##### Takotsubo Cardiomyopathy

TCM is a clinical syndrome characterized by LV dilatation and acute systolic HF, usually following an emotion or physical stressor, predominantly affecting women in the sixth decade of life. Catecholamine-mediated toxicity seems to play a key role in the pathogenesis. Echocardiography shows ballooning of the LV apex or mid-ventricle. ECG abnormalities include ST-segment elevation and T wave inversion and are accompanied by cardiac marker elevation. The therapy is supportive, and the functional recovery usually occurs within 21 days (134).

A recent study (146) reported 38 case reports of TCM, with 10 and five involving, respectively, 5-FU and capecitabine. Less frequently, TCM has been caused by antimetabolites, alkylating agents, monoclonal antibodies, and tyrosine kinase inhibitors.

TCM associated with cancer therapy affects men and women in a similar measure and occurs during the first cycle.

In hemodynamically unstable patients, the discriminating factor is the presence of LV outflow tract obstruction, as it contraindicates the use of inotropes in favor of fluids and beta-blockers. Goal-directed therapy for HF, with ACE inhibitors and beta-blocker, represents the therapeutic choice for stable patients until they recover.

##### Acute-on-Chronic Heart Failure and Advanced Heart Failure Therapies for Chemotherapy-Induced Cardiomyopathy Patients

The majority of late cardiac toxicity causing cardiomyopathy is secondary to anthracycline therapy. Anthracycline-induced cardiotoxicity can be considered, according to recent classifications (122), as a continuum phenomenon, starting at a time of first anthracycline administration, characterized by troponin release (acute), progressing to LVEF reduction (early) and then HF (late).

Therapy- and patient-specific factors determine the incidence of late cardiac toxicity: 3–5% with 400 mg/m^2^ and 18–48% at 700 mg/m^2^, with the highest risk for patients <5 or >65 years old, with preexistent cardiac diseases or cardiovascular risk factors (147).

The analysis of United Network for Organ Sharing (UNOS) or Interagency Registry for Mechanically Assisted Circulatory Support (INTERMACS) shows that 2–3% of patients receiving advanced HF therapy suffer from CCMP (84). However, there is a significant risk that this percentage could be underestimated due to misclassification of non-ischemic dilated cardiomyopathy.

Araujo-Gutierrez et al. (145) analyzed all the referrals for advanced HF of a tertiary-care center, showing that 3.4% of patients were CCMP, but the percentage was high as 7.8% in the subset of idiopathic, non-ischemic cardiomyopathy. Though not reaching statistical significance, there was a higher percentage of patients receiving LV assist device (LVAD) as bridge to transplant (BTT). In the CCMP group, compared with other Cardiomyopathy (CMP), more patients received LVAD as BTT (10.5 vs. 4.4 vs. 5.8%, ns). The percentage of total transplanted patients (including BTT) was higher in the CCMP group [CCMP 26.3% vs. non-ischemic cardiomyopathy (NICMP) 20% vs. ischemic cardiomyopathy (ICMP) 13.9%, ns]. Survival was higher in the CCMP group than other CMP, irrespective of type of therapy (orthotopic heart transplantation (OHT) or LVAD) (93.3 vs. 84.8 vs. 73.8%, respectively, *p* = 0.0021 for 1 year, 93.3 vs. 76.2 vs. 58.3%, respectively, *p* ≤ 0.0001 for 3 years).

Oliveira et al. (148) interrogated the INTERMACS database from 2006 to 2011, identifying 75 CCMP patients over 3,812 patients implanted. Seventy-two percent were female, and there were no differences in terms of preoperative New York Heart Association (NYHA) functional class, inotropes, intra-aortic balloon pump, or ventilatory support. Less than 1% received ECMO as a BTT strategy (120).

Overall, survival of patients with CCMP was similar to that of ischemic and other NICMP patients with 1-, 2-, and 3-year survival of 73, 63, and 47%, respectively. Interestingly, CCMP patients more frequently had positive markers of RV dysfunction, and the need for RV assist device (RVAD) implantation was increased, compared with ICMP and NICMP patients (19 vs. 9.3%, *p* < 0.0001), with a net negative effect on prognosis (147).

Araujo-Gutierrez et al. (145) observed an increased incidence of RV dysfunction after LVAD implantation in their cohort of 553 patients (40 vs. 18.2% from the NICMP group and 21.5% of the ICMP group). However, no RVADs were implanted in CCMP patients.

OHT is increasingly performed in CCMP patients, because of a re-evaluation of safety issues due to concerns of malignancy recurrence after immunosuppression.

Oliveira et al. (148) analyzed the outcomes of 232 patients between 2000 and 2008. No differences in outcomes between CCMP and NICMP were noted, with similar 1-, 2-, and 3-year survival [with similar 1-, 2-, and 5-year survival (86 vs. 87%, 79 vs. 81%, and 71 vs. 74%), respectively; *p* = 0.19].

Lennemann et al. (144) showed that CCMP patients due to anthracycline toxicity had a 10-year survival curve higher than all other etiologies (HR 1.28, *p* = 0.026).

In Table 1, the studies that focused on advanced HF therapies for CCMP are summarized.

**Table 1:** Characteristics of the studies on advanced therapies for HF in CCMP.

**References**	**Year**	**Therapy**	**Patients**	**Follow-up (months)**	**Characteristics and outcomes**
Armitage et al. (135)	1990	OHT	11	18	Survival 100%
Goldstein et al. (136)	1995	OHT	11	43	1-year survival, 100% 2-year survival, 81.8%
Levitt et al. (137)	1996	OHT	14	4–165	5-year survival, 74%
Koerner et al. (138)	1997	OHT	20	2–72	Survival 60%, mean survival 35 months
Taylor et al. (139)	2000	OHT	34	120	The 1-, 3-, 5-, 7-, and 10-year actuarial survival estimates for the entire group are 77, 64, 64, 64, and 50%, respectively
Fernández-Vivancos et al. (140)	2010	OHT	12	171	1-year survival, 75% 3-year survival, 75% 5-year survival, 56%
Oliveira et al. (141)	2012	OHT	232	60	Survival (95% confidence interval) at 1 year, 86% (0.81–0.91); 3 years, 79% (0.76–0.87); and 5 years, 71% (0.73–0.85)
DePasquale et al. (142)	2012	OHT	35	120	1-year survival, 71% 5-year survival, 47% 10-year survival, 32%
Lenneman et al. (143)	2013	OHT	453	120	10-year survival versus other cardiomyopathies [HR 1.28, (95% CI: 1.03–1.59), *p* = 0.026] Intravenous inotropes, 48% Admitted to ICU, 17% Need for RVAD, 5.6%
Oliveira et al. (144)	2014	LVAD	75	36	Death, 25%; OHT, 29%; recovery, 1%; alive, 44% Need for RVAD, 19% INTERMACS 1, 20%
Araujo-Gutierrez (145)	2018	5 LVAD/5 OHT	10	36	1-year survival, 93.3% 2-year survival, 93.3%

*OHT, open heart transplantation; HF, heart failure; CCMP, chemotherapy-induced cardiomyopathy; orthotopic heart transplantation; LVAD, left ventricular assist device; RVAD, right ventricular assist device; INTERMACS, Interagency Registry for Mechanically Assisted Circulatory Support*.

### Perspective of Cardiac Intensive Care Treatment

Patients suffering from acute or acute-on-chronic severe cardiac toxicity from cancer therapy must receive the same general supportive therapy of CS.

No predefined criteria of ICU triage for admission should be used, favoring an evaluation of the single case. According to Azoulay, the critically ill cancer patient can be admitted to ICU with a full code status (*Doing everything that can be done, including cancer chemotherapy*), receiving ventilatory support, vasoactive agents, renal replacement therapy, and MCS in selected cases. Clinical situation can be re-evaluated 3–5 days after admission (*ICU trial strategy*) (117).

A full code status should be warranted in the case of newly diagnosed malignancies, acute cardiac toxicity after complete cancer remission, and clinical response undetermined or still unpredictable.

Short-term MCS for refractory CS is effective to allow time for cardiac recovery or for a full clinical evaluation for further therapies (bridge-to-decision strategy) (143).

The most advanced therapies, including long-term mechanical support or heart transplantation, are not be precluded to cancer patients. Indeed, MCS guidelines state that long-term ventricular assist devices can be considered (Class of recommendation IIb, Level of Evidence B) to allow time for transplant contraindication to be reversed as recent cancer in potential transplant candidates (149, 150).

The 2016 International Society for Heart and Lung Transplantation listing criteria for heart transplantation (151) state that in patients with preexisting neoplasms, the risk of tumor recurrence should be stratified with a cardio-oncology collaboration. Cardiac transplantation should be considered when tumor recurrence is low based on tumor type, response to therapy, and negative metastatic workup. The specific amount of time to wait to transplant after neoplasm remission will depend on the aforementioned factors, and no arbitrary time period for observation should be used (Class of Recommendation I, Level of Evidence: C).

## Conclusions

**Figure 1:**
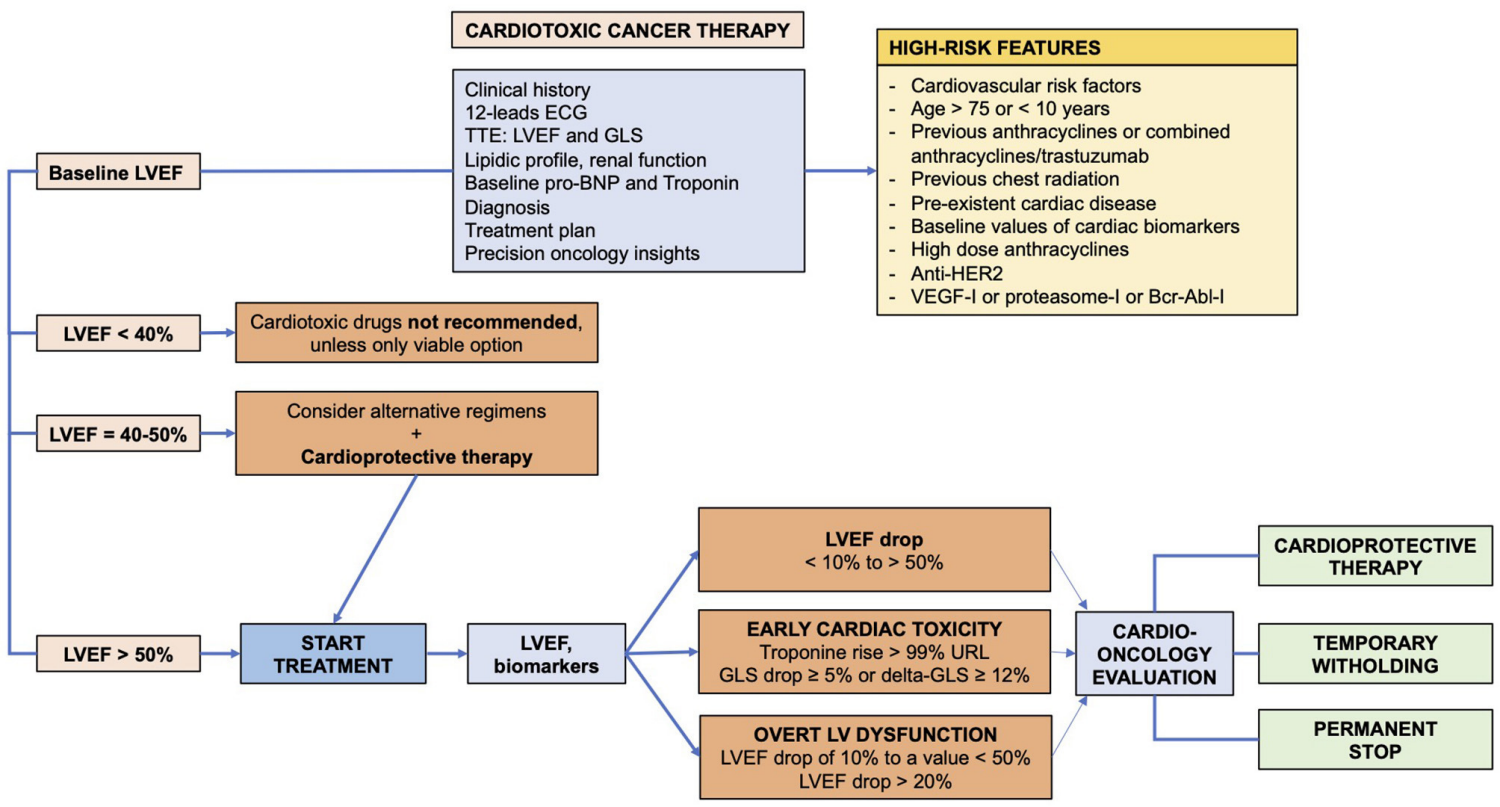
Algorithm for diagnosis and prevention of cardiac toxicity in cancer patients receiving cardiotoxic drugs.

Cardiac toxicity secondary to cancer therapies is a growing clinical issue, as the number of cancer survivors is increasing, and the number of patients developing cardiac damage will also increase. Cardiac toxicity is not limited to a single class of drugs, and it is associated even with newer biological and immunological anticancer drugs. The early recognition of toxicity should prompt therapeutic prevention and interventions, as immediate withdrawing and cardio-protective therapy (Figure 1).

For patients needing intensive care treatment, therapeutic tools are limited and not disease-modifying. Although cardio-oncology is a growing field, there are no ongoing prospective studies focusing on this subset of critically ill patients. Preventive drugs limiting cardiac damage should be tested. Besides, the increasing number of MCS implantation and OHT represents now a concrete therapy for CCMP patients with advanced HF.

## Author Contributions

Conception of the work and critical revision: AM, FD, and CN. Writing and revision: AM, MTV, VP, and JEL. Critical review: JEL and SC. All authors listed have made a substantial, direct and intellectual contribution to the work, and approved it for publication.

## Conflict of Interest

The authors declare that the research was conducted in the absence of any commercial or financial relationships that could be construed as a potential conflict of interest.

## Publisher's Note

All claims expressed in this article are solely those of the authors and do not necessarily represent those of their affiliated organizations, or those of the publisher, the editors and the reviewers. Any product that may be evaluated in this article, or claim that may be made by its manufacturer, is not guaranteed or endorsed by the publisher.

## References

[1] Herrmann J. Adverse cardiac effects of cancer therapies: cardiotoxicity and arrhythmia. Nat Rev Cardiol. (2020) 17:474–502. 10.1038/s41569-020-0348-1 32231332 PMC8782611

[2] Alexandre J Cautela J Ederhy S Damaj GL Salem JE Barlesi F. Cardiovascular toxicity related to cancer treatment: a pragmatic approach to the american and european cardio-oncology guidelines. J Am Heart Assoc. (2020) 9:e018403. 10.1161/JAHA.120.018403 32893704 PMC7727003

[3] Blum RH Carter SKA. A new anticancer drug with significant clinical activity. Ann Intern Med. (1974) 80:249–59. 4590654 10.7326/0003-4819-80-2-249

[4] Tewey KM Rowe TC Yang L Halligan BD Liu LF. Adriamycin-induced DNA damage mediated by mammalian DNA topoisomerase II. Science. (1984) 226:466–8. 10.1126/science.6093249 6093249

[5] Lyu YL Kerrigan JE Lin CP Azarova AM Tsai YC Ban Y. Topoisomerase II beta-mediated DNA double-strand breaks: implications in doxorubicin cardiotoxicity and prevention by dexrazoxane. Cancer Res. (2007) 67:8839–46. 10.1158/0008-5472.CAN-07-1649 17875725

[6] Pein F Sakiroglu O Dahan M Lebidois J Merlet P Shamsaldin A. Cardiac abnormalities 15 years and more after adriamycin therapy in 229 childhood survivors of a solid tumour at the Institut Gustave Roussy. Br J Cancer. (2004) 91:37–44. 10.1038/sj.bjc.6601904 15162142 PMC2364747

[7] Volkova M Russell R 3rd. Anthracycline cardiotoxicity: prevalence, pathogenesis and treatment. Curr Cardiol Rev. (2011) 7:214–20. 10.2174/157340311799960645 22758622 PMC3322439

[8] Von Hoff DD Layard MW Basa P Davis HL Jr Von Hoff AL Rozencweig M. Risk factors for doxorubicin-induced congestive heart failure. Ann Intern Med. (1979) 91:710–7. 10.7326/0003-4819-91-5-710 496103

[9] Fragoulakis V Roncato R Fratte CD Ecca F Bartsakoulia M Innocenti F. Estimating the effectiveness of DPYD genotyping in italian individuals suffering from cancer based on the cost of chemotherapy-induced toxicity. Am J Hum Genet. (2019) 104:1158–68. 10.1016/j.ajhg.2019.04.017 31155283 PMC6557730

[10] Meulendijks D Cats A Beijnen JH Schellens JH. Improving safety of fluoropyrimidine chemotherapy by individualizing treatment based on dihydropyrimidine dehydrogenase activity -Ready for clinical practice? Cancer Treat Rev. (2016) 50:23–34. 10.1016/j.ctrv.2016.08.002 27589829

[11] Meulendijks D Henricks LM Sonke GS Deenen MJ Froehlich TK Amstutz U. Clinical relevance of DPYD variants c.1679T>G, c.1236G>A/HapB3, and c.1601G>A as predictors of severe fluoropyrimidine-associated toxicity: a systematic review and meta-analysis of individual patient data. Lancet Oncol. (2015) 16:1639–50. 10.1016/S1470-2045(15)00286-7 26603945

[12] Yuan C Parekh H Allegra C George TJ Starr JS. 5-FU induced cardiotoxicity: case series and review of the literature. Cardiooncology. (2019) 5:13. 10.1186/s40959-019-0048-3 32154019 PMC7048125

[13] Sara JD Kaur J Khodadadi R Rehman M Lobo R Chakrabarti S. 5-fluorouracil and cardiotoxicity: a review. Ther Adv Med Oncol. (2018) 10:1758835918780140. 10.1177/1758835918780140 29977352 PMC6024329

[14] Lestuzzi C Vaccher E Talamini R Lleshi A Meneguzzo N Viel E. Effort myocardial ischemia during chemotherapy with 5-fluorouracil: an underestimated risk. Ann Oncol. (2014) 25:1059–64. 10.1093/annonc/mdu055 24558023

[15] de Forni M Malet-Martino MC Jaillais P Shubinski RE Bachaud JM Lemaire L. Cardiotoxicity of high-dose continuous infusion fluorouracil: a prospective clinical study. J Clin Oncol. (1992) 10:1795–801. 10.1200/JCO.1992.10.11.1795 1403060

[16] Çalik AN Çeliker E Velibey Y Çagdaş M Güzelburç Ö. Initial dose effect of 5-fluorouracil: rapidly improving severe, acute toxic myopericarditis. Am J Emerg Med. (2012) 30:257.e1–e3. 10.1016/j.ajem.2010.10.025 21208764

[17] Robben NC Pippas AW Moore JO. The syndrome of 5-fluorouracil cardiotoxicity. An elusive cardiopathy. Cancer. (1993) 71:493–509. 8422644 10.1002/1097-0142(19930115)71:2<493::aid-cncr2820710235>3.0.co;2-c

[18] Alter P Herzum M Soufi M Schaefer JR Maisch B. Cardiotoxicity of 5-fluorouracil. Cardiovasc Hematol Agents Med Chem. (2006) 4:1–5. 10.2174/187152506775268785 16529545

[19] Burger AJ Mannino S. 5-Fluorouracil-induced coronary vasospasm. Am Heart J. (1987) 114:433–6. 3604903 10.1016/0002-8703(87)90517-5

[20] Südhoff T Enderle MD Pahlke M Petz C Teschendorf C Graeven U. 5-Fluorouracil induces arterial vasocontractions. Ann Oncol. (2004) 15:661–4. 10.1093/annonc/mdh150 15033676

[21] Heistad DD Armstrong ML Marcus ML Piegors DJ Mark AL. Augmented responses to vasoconstrictor stimuli in hypercholesterolemic and atherosclerotic monkeys. Circ Res. (1984) 54:711–8. 10.1161/01.RES.54.6.711 6733866

[22] Lopez JA Armstrong ML Piegors DJ Heistad DD. Effect of early and advanced atherosclerosis on vascular responses to serotonin, thromboxane A2, and ADP. Circulation. (1989) 79:698–705. 10.1161/01.CIR.79.3.698 2917393

[23] Henry PD Yokoyama M. Supersensitivity of atherosclerotic rabbit aorta to ergonovine. Mediation by a serotonergic mechanism. J Clin Invest. (1980) 66:306–13. 10.1172/JCI109858 7400317 PMC371712

[24] Kinhult S Albertsson M Eskilsson J Cwikiel M. Antithrombotic treatment in protection against thrombogenic effects of 5-fluorouracil on vascular endothelium: a scanning microscopy evaluation. Scanning. (2001) 23:1–8. 10.1002/sca.4950230101 11272331

[25] Jensen SA Sørensen JB. 5-fluorouracil-based therapy induces endovascular injury having potential significance to development of clinically overt cardiotoxicity. Cancer Chemother Pharmacol. (2012) 69:57–64. 10.1007/s00280-011-1669-x 21603868

[26] Schalkwijk CG Poland DC van Dijk W Kok A Emeis JJ Dräger AM. Plasma concentration of C-reactive protein is increased in type I diabetic patients without clinical macroangiopathy and correlates with markers of endothelial dysfunction: evidence for chronic inflammation. Diabetologia. (1999) 42:351–7. 10.1007/s001250051162 10096789

[27] Gershwin ME Goetzl EJ Steinberg AD. Cyclophosphamide: use in practice. Ann Intern Med. (1974) 80:531–40. 10.7326/0003-4819-80-4-531 4621265

[28] Field JJ Kanakkanthara A Miller JH. Microtubule-targeting agents are clinically successful due to both mitotic and interphase impairment of microtubule function. Bioorg Med Chem. (2014) 22:5050–9. 10.1016/j.bmc.2014.02.035 24650703

[29] Giordano SH Booser DJ Murray JL Ibrahim NK Rahman ZU Valero V. A detailed evaluation of cardiac toxicity: a phase II study of doxorubicin and one- or three-hour-infusion paclitaxel in patients with metastatic breast cancer. Clin Cancer Res. (2002) 8:3360–8. 12429622

[30] Gianni L Baselga J Eiermann W Guillem Porta V Semiglazov V Lluch A. Feasibility and tolerability of sequential doxorubicin/paclitaxel followed by cyclophosphamide, methotrexate, and fluorouracil and its effects on tumor response as preoperative therapy. Clin Cancer Res. (2005) 11:8715–21. 10.1158/1078-0432.CCR-05-0539 16361558

[31] Napoli C Benincasa G Donatelli F Ambrosio G. Precision medicine in distinct heart failure phenotypes: focus on clinical epigenetics. Am Heart J. (2020) 224:113–28. 10.1016/j.ahj.2020.03.007 32361531

[32] Pritchard KI Shepherd LE O'Malley FP Andrulis IL Tu D Bramwell VH. HER2 and responsiveness of breast cancer to adjuvant chemotherapy. N Engl J Med. (2006) 354:2103–11. 10.1056/NEJMoa054504 16707747

[33] Goldenberg MM. Trastuzumab, a recombinant DNA-derived humanized monoclonal antibody, a novel agent for the treatment of metastatic breast cancer. Clin Ther. (1999) 21:309–18. 10211534 10.1016/S0149-2918(00)88288-0

[34] Piccart-Gebhart MJ Procter M Leyland-Jones B Goldhirsch A Untch M Smith I. Trastuzumab after adjuvant chemotherapy in HER2-positive breast cancer. N Engl J Med. (2005) 353:1659–72. 10.1056/NEJMoa052306 16236737

[35] Sarno F Benincasa G List M Barabasi AL Baumbach J Ciardiello F. Clinical epigenetics settings for cancer and cardiovascular diseases: real-life applications of network medicine at the bedside. Clin Epigenetics. (2021) 13:66. 10.1186/s13148-021-01047-z 33785068 PMC8010949

[36] Monsuez JJ Charniot JC Vignat N Artigou JY. Cardiac side-effects of cancer chemotherapy. Int J Cardiol. (2010) 144:3–15. 10.1016/j.ijcard.2010.03.003 20399520

[37] Bowles EJ Wellman R Feigelson HS Onitilo AA Freedman AN Delate T. Risk of heart failure in breast cancer patients after anthracycline and trastuzumab treatment: a retrospective cohort study. J Natl Cancer Inst. (2012) 104:1293–305. 10.1093/jnci/djs317 22949432 PMC3433392

[38] Gradishar WJ Anderson BO Abraham J Aft R Agnese D Allison KH. Breast cancer, version 3.2020, NCCN clinical practice guidelines in oncology. J Natl Compr Canc Netw. (2020) 18:452–78. 10.6004/jnccn.2020.0016 32259783

[39] Valachis A Nearchou A Lind P Mauri D. Lapatinib, trastuzumab or the combination added to preoperative chemotherapy for breast cancer: a meta-analysis of randomized evidence. Breast Cancer Res Treat. (2012) 135:655–62. 10.1007/s10549-012-2189-z 22875745

[40] Krop IE Suter TM Dang CT Dirix L Romieu G Zamagni C. Feasibility and cardiac safety of trastuzumab emtansine after anthracycline-based chemotherapys (neo) adjuvant therapy for human epidermal growth factor receptor 2-positive early-stage breast cancer. J Clin Oncol. (2015) 33:1136–42. 10.1200/JCO.2014.58.7782 25713436 PMC5657012

[41] Ferrara N. Role of vascular endothelial growth factor in regulation of physiological angiogenesis. Am J Physiol Cell Physiol. (2001) 280:C1358–66. 10.1152/ajpcell.2001.280.6.C1358 11350730

[42] Yang B Papoian T. Tyrosine kinase inhibitor (TKI)-induced cardiotoxicity: approaches to narrow the gaps between preclinical safety evaluation and clinical outcome. J Appl Toxicol. (2012) 32:945–51. 10.1002/jat.2813 22961481

[43] De Pascale MR Della Mura N Vacca M Napoli C. Useful applications of growth factors for cardiovascular regenerative medicine. Growth Factors. (2020) 38:35–63. 10.1080/08977194.2020.1825410 33028111

[44] Groarke JD Choueiri TK Slosky D Cheng S Moslehi J. Recognizing and managing leftventricular dysfunction associated with therapeutic inhibition of the vascular endothelial growth factor signaling pathway. CurrTreat Options Cardiovasc Med. (2014) 16:335. 10.1007/s11936-014-0335-0 25099086

[45] Nazer B Humphreys BD Moslehi J. Effects of novel angiogenesis inhibitors for the treatment of cancer on the cardiovascular system: focus on hypertension. Circulation. (2011) 124:1687–91. 10.1161/CIRCULATIONAHA.110.992230 21986775

[46] Youn JY Wang T Cai H. An ezrin/calpain/PI3K/AMPK/ eNOSs1179 signaling cascade mediating VEGFdependent endothelial nitric oxide production. Circ Res. (2009) 104:50–9. 10.1161/CIRCRESAHA.108.178467 19038867 PMC2720268

[47] Mourad JJ Levy BI. Mechanisms of antiangiogenicinduced arterial hypertension. Curr Hypertens Rep. (2011) 13:289–93. 10.1007/s11906-011-0206-y 21479992

[48] Steeghs N Rabelink TJ op 't Roodt J Batman E Cluitmans FH Weijl NI. Reversibility of capillary density after discontinuation of bevacizumab treatment. Ann Oncol. (2010) 21:1100–5. 10.1093/annonc/mdp417 19854721

[49] Vigneau C Lorcy N Dolley-Hitze T Jouan F Arlot-Bonnemains Y Laguerre B. All antivascular endothelial growth factor drugs can induce ‘pre-eclampsia-like syndrome': a RARe study. Nephrol Dial Transplant. (2014) 29:325–32. 10.1093/ndt/gft465 24302609

[50] Brinda BJ Viganego F Vo T Dolan D Fradley MG. Anti-VEGF-induced hypertension: a review of pathophysiology and treatment options. Curr Treat Options Cardiovasc Med. (2016) 18:33. 10.1007/s11936-016-0452-z 26932588

[51] Hall PS Harshman LC Srinivas S Witteles RM. The frequency and severity of cardiovascular toxicity from targeted therapy in advanced renal cell carcinoma patients. JACC Heart Fail. (2013) 1:72–8. 10.1016/j.jchf.2012.09.001 24621801

[52] Lichtenstein D van Hooland S Elbers P Malbrain ML. Ten good reasons to practice ultrasound in critical care. Anaesthesiol Intensive Ther. (2014) 46:323–35. 10.5603/AIT.2014.0056 25432552

[53] Plana JC Galderisi M Barac A Ewer MS Ky B Scherrer-Crosbie M. Expert consensus for multimodality imaging evaluation of adult patients during and after cancer therapy: a report from the American Society of Echocardiography and the European Association of Cardiovascular Imaging. Eur Heart J Cardiovasc Imaging. (2014) 15:1063–93. 10.1093/ehjci/jeu192 25239940 PMC4402366

[54] Lang RM Bierig M Devereux RB Flachskampf FA Foster E Pellikka PA. Recommendations for chamber quantification: a report from the American Society of Echocardiography's Guidelines and Standards Committee and the Chamber Quantification Writing Group, developed in conjunction with the European Association of Echocardiography, a branch of the European Society of Cardiology. J Am Soc Echocardiogr. (2005) 18:1440–63. 10.1016/j.echo.2005.10.005 16376782

[55] Maja Cikes Scott DS. Beyond ejection fraction: an integrative approach for assessment of cardiac structure and function in heart failure. Eur Heart J. (2016) 37:1642–50. 10.1093/eurheartj/ehv510 26417058

[56] Plana JC Galderisi M Barac A Ewer MS Ky B Scherrer-Crosbie M. Expert consensus for multimodality imaging evaluation of adult patients during and after cancer therapy: a report from the American Society of Echocardiography and the European Association of Cardiovascular Imaging. J Am Soc Echocardiogr. (2014) 27:911–39. 10.1016/j.echo.2014.07.012 25172399

[57] Thavendiranathan P Grant AD Negishi T Plana JC Popović ZB Marwick TH. Reproducibility of echocardiographic techniques for sequential assessment of left ventricular ejection fraction and volumes: application to patients undergoing cancer chemotherapy. J Am Coll Cardiol. (2013) 61:77–84. 10.1016/j.jacc.2012.09.035 23199515

[58] Liu J Banchs J Mousavi N Plana JC Scherrer-Crosbie M Thavendiranathan P. Contemporary role of echocardiography for clinical decision making in patients during and after cancer therapy. JACC Cardiovasc Imaging. (2018) 11:1122–31. 10.1016/j.jcmg.2018.03.025 30092969

[59] Sawaya H Sebag IA Plana JC Januzzi JL Ky B Tan TC. Assessment of echocardiography and biomarkers for the extended prediction of cardiotoxicity in patients treated with anthracyclines, taxanes, and trastuzumab. Circ Cardiovasc Imaging. (2012) 5:596–603. 10.1161/CIRCIMAGING.112.973321 22744937 PMC3703313

[60] Stoodley PW Richards DA Boyd A Hui R Harnett PR Meikle SR. Left ventricular systolic function in HER2/neu negative breast cancer patients treated with anthracycline chemotherapy: a comparative analysis of leftventricular ejection fraction and myocardial strain imaging over 12 months. Eur J Cancer. (2013) 49:3396–403. 10.1016/j.ejca.2013.06.046 23937961

[61] Negishi K Negishi T Hare JL Haluska BA Plana JC Marwick TH. Independent and incremental value of deformation indices for prediction of trastuzumab-induced cardiotoxicity. J Am Soc Echocardiogr. (2013) 26:493–8. 10.1016/j.echo.2013.02.008 23562088

[62] Clasen SC Scherrer-Crosbie M. Applications of left ventricular strain measurements to patients undergoing chemotherapy. Curr Opin Cardiol. (2018) 33:493–7. 10.1097/HCO.0000000000000541 30028729

[63] Oreto L Todaro MC Umland MM Kramer C Qamar R Carerj S. Use of echocardiography to evaluate the cardiac effects of therapies used in cancer treatment: what do we know? J Am Soc Echocardiogr. (2012) 25:1141–52. 10.1016/j.echo.2012.09.001 23000452

[64] Stoddard MF Seeger J Liddell NE Hadley TJ Sullivan DM Kupersmith J. Prolongation of isovolumetric relaxation time as assessed by Doppler echocardiography predicts doxorubicin-induced systolic dysfunction in humans. J Am Coll Cardiol. (1992) 20:62–9. 10.1016/0735-1097(92)90138-D 1607540

[65] Tassan-Mangina S Codorean D Metivier M Costa B Himberlin C Jouannaud C. Tissue Doppler imaging and conventional echocardiography after anthracycline treatment in adults: early and late alterations of left ventricular function during a prospective study. Eur J Echocardiogr. (2006) 7:141–6. 10.1016/j.euje.2005.04.009 15941672

[66] Mason JW Bristow MR Billingham ME Daniels JR. Invasive and noninvasive methods of assessing adriamycin cardiotoxic effects in man: superiority of histopathologic assessment using endomyocardial biopsy. Cancer Treat Rep. (1978) 62:857–64. 667859

[67] Tanindi A Demirci U Tacoy G Buyukberber S Alsancak Y Coskun U. Assessment of right ventricular functions during cancer chemotherapy. Eur J Echocardiogr. (2011) 12:834–40. 10.1093/ejechocard/jer142 21880609

[68] Sugimoto T. Acute decompensated heart failure in patients with heart failure with preserved ejection fraction. Heart Fail Clin. (2020) 16:201–9. 10.1016/j.hfc.2019.12.002 32143764

[69] Weeks SG Shapiro M Foster E Michaels AD. Echocardiographic predictors of change in left ventricular diastolic pressure in heart failure patients receiving nesiritide. Echocardiography. (2008) 25:849–55. 10.1111/j.1540-8175.2008.00705.x 18986412

[70] Arques S Roux E Sbragia P Ambrosi P Taieb L Pieri B. Accuracy of tissue Doppler echocardiography in the emergency diagnosis of decompensated heart failure with preserved left ventricular systolic function: comparison with B-type natriuretic peptide measurement. Echocardiography. (2005) 22:657–64. 10.1111/j.1540-8175.2005.40076.x 16174119

[71] Rudas M Orde S Nalos M. Bedside lung ultrasound in the care of the critically ill. Crit Care Resusc. (2017) 19:327–36. 29202259

[72] Mukai-Yatagai N Haruki N Kinugasa Y Ohta Y Ishibashi-Ueda H Akasaka T. Assessment of myocardial fibrosis using T1-mapping and extracellular volume measurement on cardiac magnetic resonance imaging for the diagnosis of radiation-induced cardiomyopathy. J Cardiol Cases. (2018) 18:132–5. 10.1016/j.jccase.2018.06.001 30279930 PMC6149610

[73] Biersmith MA Tong MS Guha A Simonetti OP Addison D. Multimodality cardiac imaging in the era of emerging cancer therapies. J Am Heart Assoc. (2020) 9:e013755. 10.1161/JAHA.119.013755 31960741 PMC7033826

[74] Mahrholdt H Wagner A Judd RM Sechtem U. Assessment of myocardial viability by cardiovascular magnetic resonance imaging. Eur Heart J. (2002) 23:602–19. 10.1053/euhj.2001.3038 11969275

[75] Mahrholdt H Goedecke C Wagner A Meinhardt G Athanasiadis A Vogelsberg H. Cardiovascular magnetic resonance assessment of human myocarditis: a comparison to histology and molecular pathology. Circulation. (2004) 109:1250–8. 10.1161/01.CIR.0000118493.13323.81 14993139

[76] Mahmood SS Fradley MG Cohen JV Nohria A Reynolds KL Heinzerling LM. Myocarditis in patients treated with immune checkpoint inhibitors. J Am Coll Cardiol. (2018) 71:1755–64. 10.1016/j.jacc.2018.02.037 29567210 PMC6196725

[77] Infante T Forte E Schiano C Punzo B Cademartiri F Cavaliere C. Evidence of association of circulating epigenetic-sensitive biomarkers with suspected coronary heart disease evaluated by cardiac computed tomography. PLoS ONE. (2019) 14:e0210909. 10.1371/journal.pone.0210909 30673762 PMC6343931

[78] Detrano R Guerci AD Carr JJ Bild DE Burke G Folsom AR. Coronary calcium as a predictor of coronary events in four racial or ethnic groups. N Engl J Med. (2008) 358:1336–45. 10.1056/NEJMoa072100 18367736

[79] Osborne MT Hulten EA Singh A Waller AH Bittencourt MS Stewart GC. Reduction in ^18^F-fluorodeoxyglucose uptake on serial cardiac positron emission tomography associated with improved left ventricular ejection fraction in patients with cardiac sarcoidosis. J Nucl Cardiol. (2014) 21:166–74. 10.1007/s12350-013-9828-6 24307261

[80] Wang J Hu Y Yang S Wei G Zhao X Wu W. Role of fluorodeoxyglucose positron emission tomography/computed tomography in predicting the adverse effects of chimeric antigen receptor T cell therapy in patients with non-Hodgkin lymphoma. Biol Blood Marrow Transplant. (2019) 25:1092–8. 10.1016/j.bbmt.2019.02.008 30769193

[81] Benincasa G Mansueto G Napoli C. Fluid-based assays and precision medicine of cardiovascular diseases: the 'hope' for Pandora's box? J Clin Pathol. (2019) 72:785–99. 10.1136/jclinpath-2019-206178 31611285

[82] Pudil R Mueller C Celutkiene J Henriksen PA Lenihan D Dent S. Role of serum biomarkers in cancer patients receiving cardiotoxic cancer therapies: a position statement from the Cardio-Oncology Study Group of the Heart Failure Association and the Cardio-Oncology Council of the European Society of Cardiology. Eur J Heart Fail. (2020) 22:1966–83. 10.1002/ejhf.2017 33006257

[83] Omland T de Lemos JA Sabatine MS Christophi CA Rice MM Jablonski KA de Lemos J A. A sensitive cardiac troponin T assay in stable coronary artery disease. N Engl J Med. (2009) 361:2538–47. 10.1056/NEJMoa0805299 19940289 PMC2997684

[84] Cardinale D Sandri MT Colombo A Colombo N Boeri M Lamantia G. Prognostic value of troponin I in cardiac risk stratification of cancer patients undergoing high-dose chemotherapy. Circulation. (2004) 109:2749–54. 10.1161/01.CIR.0000130926.51766.CC 15148277

[85] Cardinale D Colombo A Torrisi R Sandri MT Civelli M Salvatici M. Trastuzumab-induced cardiotoxicity: clinical and prognostic implications of troponin I evaluation. J Clin Oncol. (2010) 28:3910–6. 10.1200/JCO.2009.27.3615 20679614

[86] Michel L Mincu RI Mahabadi AA Settelmeier S Al-Rashid F Rassaf T. Troponins and brain natriuretic peptides for the prediction of cardiotoxicity in cancer patients: a meta-analysis. Eur J Heart Fail. (2020) 22:350–61. 10.1002/ejhf.1631 31721381

[87] Tzolos E Adamson PD Hall PS Macpherson IR Oikonomidou O MacLean M. Dynamic changes in high-sensitivity cardiac troponin i in response to anthracycline-based chemotherapy. Clin Oncol. (2020) 32:292–7. 10.1016/j.clon.2019.11.008 31813662 PMC7139216

[88] Shafi A Siddiqui N Imtiaz S Din Sajid MU. Left ventricular systolic dysfunction predicted by early troponin I release after anthracycline based chemotherapy in breast cancer patients. J Ayub Med College Abbottabad. (2017) 29:266–9. 28718245

[89] Olivieri J Perna GP Bocci C Montevecchi C Olivieri A Leoni P. Modern management of anthracycline-induced cardiotoxicity in lymphoma patients: low occurrence of cardiotoxicity with comprehensive assessment and tailored substitution by nonpegylated liposomal doxorubicin. Oncologist. (2017) 22:422–31. 10.1634/theoncologist.2016-0289 28275118 PMC5388379

[90] Ky B Putt M Sawaya H French B Januzzi JL Jr Sebag IA. Early increases in multiple biomarkers predict subsequent cardiotoxicity in patients with breast cancer treated with doxorubicin, taxanes, and trastuzumab. J Am Coll Cardiol. (2014) 63:809–16. 10.1016/j.jacc.2013.10.061 24291281 PMC4286181

[91] Cardinale D Sandri MT Martinoni A Tricca A Civelli M Lamantia G. Left ventricular dysfunction predicted by early troponin I release after high-dose chemotherapy. J Am College Cardiol. (2000) 36:517–22. 10.1016/S0735-1097(00)00748-8 10933366

[92] Ananthan K Lyon AR. The Role of Biomarkers in Cardio-Oncology. J Cardiovasc Transl Res. (2020) 13:431–50. 10.1007/s12265-020-10042-3 32642841 PMC7360533

[93] Pourier MS Kapusta L van Gennip A Bökkerink JP Loonen J Bellersen L. Values of high sensitive troponin T in long-term survivors of childhood cancer treated with anthracyclines. Clinica Chimica Acta. (2015) 441:29–32. 10.1016/j.cca.2014.12.011 25512165

[94] Ylänen K Poutanen T Savukoski T Eerola A Vettenranta K. Cardiac biomarkers indicate a need for sensitive cardiac imaging among long-term childhood cancer survivors exposed to anthracyclines. Acta Paediatrica. (2015) 104:313–9. 10.1111/apa.12862 25393922

[95] Ceyhan C Meydan N Barutca S Tekten T Onbasili AO Ozturk B. Influence of high-dose leucovorin and 5- fluorouracil chemotherapy regimen on P wave duration and dispersion. J Clin Phar. Ther. (2004) 29:267–71. 10.1111/j.1365-2710.2004.00556.x 15153089

[96] Turan T Agac MT Aykan AÇ Kul S Akyüz AR Gökdeniz T. Usefulness of heart-type fatty acid-binding protein and myocardial performance index for early detection of 5-fluorouracil cardiotoxicity. Angiology. (2017) 68:52–8. 10.1177/0003319716637516 26980771

[97] Oztop I Gencer M Okan T Yaren A Altekin E Turker S. Evaluation of cardiotoxicity of a combined bolus plus infusional 5-fluorouracil/folinic acid treatment by echocardiography, plasma troponin I level, QT interval and dispersion in patients with gastrointestinal system cancers. Jpn J Clin Oncol. (2004) 34:262–8. 10.1093/jjco/hyh047 15231861

[98] Salepci T Seker M Uyarel H Gumus M Bilici A Ustaalioglu BBSeker M. 5-Fluorouracil induces arterial vasoconstrictions but does not increase angiotensin II levels. Med Oncol. (2010) 27:416–20. 10.1007/s12032-009-9226-8 19415535

[99] Holubec L Jr Topolcan O Finek J Salvet J Svoboda T Svobodova S. Dynamic monitoring of cardio-specific markers and markers of thyroid gland function in cancer patients–a pilot study. Anticancer Res. (2007) 27:1883–6. 17649788

[100] Curigliano G Cardinale D Suter T Plataniotis G de Azambuja E Sandri MT. Cardiovascular toxicity induced by chemotherapy, targeted agents and radiotherapy: ESMO clinical practice guidelines. Ann Oncol. (2012) 23 (Suppl. 7):vii155–66. 10.1093/annonc/mds293 22997448

[101] Ederhy S Massard C Dufaitre G Balheda R Meuleman C Rocca CG. Frequency and management of troponin I elevation in patients treated with molecular targeted therapies in phase I trials. Invest New Drugs. (2012) 30:611–5. 10.1007/s10637-010-9546-8 20924643

[102] Johnson DB Balko JM Compton ML Chalkias S Gorham J Xu Y. Fulminant myocarditis with combination immune checkpoint blockade. N Engl J Med. (2016) 375:1749–55. 10.1056/NEJMoa1609214 27806233 PMC5247797

[103] Dodos F Halbsguth T Erdmann E Hoppe UC. Usefulness of myocardial performance index and biochemical markers for early detection of anthracycline-induced cardiotoxicity in adults. Clin Res Cardiol. (2008) 97:318–26. 10.1007/s00392-007-0633-6 18193371

[104] De Iuliis F Salerno G Taglieri L De Biase L Lanza R Cardelli P. Serum biomarkers evaluation to predict chemotherapy-induced cardiotoxicity in breast cancer patients. Tumor Biol. (2016) 37:3379–87. 10.1007/s13277-015-4183-7 26449821

[105] Lenihan DJ Stevens PL Massey M Plana JC Araujo DM Fanale MA. The utility of point-of-care biomarkers to detect cardiotoxicity during anthracycline chemotherapy: a feasibility study. J Cardiac Fail. (2016) 22:433–8. 10.1016/j.cardfail.2016.04.003 27079675

[106] Zardavas D Suter TM Van Veldhuisen DJ Steinseifer J Noe J Lauer S. Role of troponins I and T and N-terminal prohormone of brain natriuretic peptide in monitoring cardiac safety of patients with early-stage human epidermal growth factor receptor 2-positive breast cancer receiving trastuzumab: a herceptin adjuvant study cardiac marker substudy. J Clin Oncol. (2017) 35:878–84. 10.1200/JCO.2015.65.7916 28199174

[107] Palumbo I Palumbo B Fravolini ML Marcantonini M Perrucci E Latini ME. Brain natriuretic peptide as a cardiac marker of transient radiotherapy related damage in left-sided breast cancer patients: a prospective study. Breast. (2016) 25:45–50. 10.1016/j.breast.2015.10.004 26547836

[108] Jensen SA Hasbak P Mortensen J Sørensen JB. Fluorouracil induces myocardial ischemia with increases of plasma brain natriuretic peptide and lactic acid but without dysfunction of left ventricle. J Clin Oncol. (2010) 28:5280–6. 10.1200/JCO.2009.27.3953 21079148

[109] Ridker PM Lüscher TF. Anti-inflammatory therapies for cardiovascular disease. Eur Heart J. (2014) 35:1782–91. 10.1093/eurheartj/ehu203 24864079 PMC4155455

[110] Morris PG Chen C Steingart R Fleisher M Lin N Moy B. Troponin I and C-reactive protein are commonly detected in patients with breast cancer treated with dosedense chemotherapy incorporating trastuzumab and lapatinib. Clin Cancer Res. (2011) 17:3490–9. 10.1158/1078-0432.CCR-10-1359 21372222

[111] Fallah-Rad N Walker JR Wassef A Lytwyn M Bohonis S Fang T. The utility of cardiac biomarkers, tissue velocity and strain imaging, and cardiac magnetic resonance imaging in predicting early left ventricular dysfunction in patients with human epidermal growth factor receptor II–positive breast cancer treated with adjuvant trastuzumab therapy. J Am College Cardiol. (2011) 57:2263–70. 10.1016/j.jacc.2010.11.063 21616287

[112] Onitilo AA Engel JM Stankowski RV Liang H Berg RL Doi SA. High-sensitivity C-reactive protein (hs-CRP) as a biomarker for trastuzumab-induced cardiotoxicity in HER2-positive early-stage breast cancer: a pilot study. Breast Cancer Res Treat. (2012) 134:291–8. 10.1007/s10549-012-2039-z 22476854

[113] Depetris I Marino D Bonzano A Cagnazzo C Filippi R Aglietta M. Fluoropyrimidine-induced cardiotoxicity. Crit Rev Oncol Hematol. (2018) 124:1–10. 10.1016/j.critrevonc.2018.02.002 29548480

[114] Reichlin T Socrates T Egli P Potocki M Breidthardt T Arenja N. Use of myeloperoxidase for risk stratification in acute heart failure. Clin Chem. (2010) 56:944–51. 10.1373/clinchem.2009.142257 20413430

[115] Bos MM Verburg IW Dumaij I Stouthard J Nortier JW Richel DVerburg IW. Intensive care admission of cancer patients: a comparative analysis. Cancer Med. (2015) 4:966–76. 10.1002/cam4.430 25891471 PMC4529335

[116] Shimabukuro-Vornhagen A Boll B Kochanek M Azoulay E von Bergwelt-Baildon MS. Critical care of patients with cancer. CA Cancer J Clin. (2016) 66:496–517. 10.3322/caac.21351 27348695

[117] Azoulay E Schellongowski P Darmon M Bauer PR Benoit D Depuydt P. The intensive care medicine research agenda on critically ill oncology and hematology patients. Intensive Care Med. (2017) 43:1366–82. 10.1007/s00134-017-4884-z 28725926

[118] Crimi E Benincasa G Figueroa-Marrero N Galdiero M Napoli C. Epigenetic susceptibility to severe respiratory viral infections and its therapeutic implications: a narrative review. Br J Anaesth. (2020) 125:1002–17. 10.1016/j.bja.2020.06.060 32828489 PMC7438995

[119] Puymirat E Fagon JY Aegerter P Diehl JL Monnier A Hauw-Berlemont C. Cardiogenic shock in intensive care units: evolution of prevalence, patient profile, management and outcomes, 1997-2012. Eur J Heart Fail. (2017) 19:192–200. 10.1002/ejhf.646 27709722

[120] Montisci A Donatelli F Cirri S Coscioni E Maiello C Napoli C. Veno-arterial extracorporeal membrane oxygenation as bridge to heart transplantation: the way forward. Transplant Direct. (2021) 7:e720. 10.1097/TXD.0000000000001172 34258387 PMC8270578

[121] Ma WW Saif MW El-Rayes BF Fakih MG Cartwright TH Posey JA. Emergency use of uridine triacetate for the prevention and treatment of life-threatening 5-fluorouracil and capecitabine toxicity. Cancer. (2017) 123:345–56. 10.1002/cncr.30321 27622829 PMC5248610

[122] Cardinale D Iacopo F Cipolla CM. Cardiotoxicity of anthracyclines. Front Cardiovasc Med. (2020) 7:26. 10.3389/fcvm.2020.00026 32258060 PMC7093379

[123] Rudzinski T Ciesielczyk M Religa W Bednarkiewicz Z Krzeminska-Pakula M. Doxorubicin-induced ventricular arrhythmia treated by implantation of an automatic cardioverter-defibrillator. Europace. (2007) 9:278–80. 10.1093/europace/eum033 17383986

[124] Wouters KA Kremer LC Miller TL Herman EH Lipshultz SE. Protecting against anthracycline-induced myocardial damage: a review of the most promising strategies. Br J Haematol. (2005) 131:561–78. 10.1111/j.1365-2141.2005.05759.x 16351632

[125] Cowgill JA Francis SA Sawyer DB. Anthracycline and peripartum cardiomyopathies. Circ Res. (2019) 124:1633–46. 10.1161/CIRCRESAHA.119.313577 31120822

[126] Polk A Vaage-Nilsen M Vistisen K Nielsen DL. Cardiotoxicity in cancer patients treated with 5-fluorouracil or capecitabine: a systematic review of incidence, manifestations and predisposing factors. Cancer Treat Rev. (2013) 39:974–84. 10.1016/j.ctrv.2013.03.005 23582737

[127] Jensen SA Sørensen JB. Risk factors and prevention of cardiotoxicity induced by 5-fluorouracil or capecitabine. Cancer Chemother Pharmacol. (2006) 58:487–93. 10.1007/s00280-005-0178-1 16418875

[128] Jerusalem G Lancellotti P Kim SB. HER2+ breast cancer treatment and cardiotoxicity: monitoring and management. Breast Cancer Res Treat. (2019) 177:237–50. 10.1007/s10549-019-05303-y 31165940 PMC6661020

[129] Minichillo S Gallelli I Barbieri E Cubelli M Rubino D Quercia S. Trastuzumab resumption after extremely severe cardiotoxicity in metastatic breast cancer patient: a case report. BMC Cancer. (2017) 17:722. 10.1186/s12885-017-3712-8 29115937 PMC5678795

[130] Escudier M Cautela J Malissen N Ancedy Y Orabona M Pinto J. Clinical features, management, and outcomes of immune checkpoint inhibitor-related cardiotoxicity. Circulation. (2017) 136:2085–2087. 10.1161/CIRCULATIONAHA.117.030571 29158217

[131] Moslehi J Lichtman AH Sharpe AH Galluzzi L Kitsis RN. Immune checkpoint inhibitor-associated myocarditis: manifestations and mechanisms. J Clin Invest. (2021) 131:e145186. 10.1172/JCI145186 33645548 PMC7919710

[132] Ganatra S Neilan TG. Immune checkpoint inhibitor-associated myocarditis. Oncologist. (2018) 23:879–886. 10.1634/theoncologist.2018-0130 29802219 PMC6156176

[133] Brahmer JR Lacchetti C Schneider BJ Atkins MB Brassil KJ Caterino JM. Management of immune-related adverse events in patients treated with immune checkpoint inhibitor therapy: American Society of Clinical Oncology Clinical Practice Guideline. J Clin Oncol. (2018) 36:1714–68. 10.1200/JCO.2017.77.6385 29442540 PMC6481621

[134] Ghadri JR Wittstein IS Prasad A Sharkey S Dote K Akashi YJ. International expert consensus document on takotsubo syndrome (part i): clinical characteristics, diagnostic criteria, and pathophysiology. Eur Heart J. (2018) 39:2032–46. 10.1093/eurheartj/ehy076 29850871 PMC5991216

[135] Armitage JM Kormos RL Griffith BP Fricker FJ Hardesty RL. Heart transplantation in patients with malignant disease. J Heart Transplant. (1990) 9:627–9. 2277299

[136] Goldstein DJ Seldomridge JA Addonizio L Rose EA Oz MC Michler RE. Orthotopic heart transplantation in patients with treated malignancies. Am J Cardiol. (1995) 75:968–71. 10.1016/S0002-9149(99)80704-8 7733018

[137] Levitt G Bunch K Rogers CA Whitehead B. Cardiac transplanta- tion in childhood cancer survivors in Great Britain. Eur J Cancer. (1996) 32A:826–30. 10.1016/0959-8049(96)00028-7 9081361

[138] Koerner MM Tenderich G Minami K Mannebach H Koertke H zu Knyphausen E. Results of heart transplantation in patients with pre- existing malignancies. Am J Cardiol. (1997) 79:988–91. 10.1016/S0002-9149(97)00031-3 9104923

[139] Taylor DO Farhoud HH Kfoury G Pham SM Pigula FA Kormos RL. Cardiac transplantation in survivors of lymphoma: a multi- institutional survey. Transplantation. (2000) 69:2112–5. 10.1097/00007890-200005270-00025 10852607

[140] Fernández-Vivancos C Paniagua-Martín MJ Marzoa-Rivas R Barge-Caballero E Grile-Cancela Z Recio-Mayoral A. Long-term outcome in heart transplant patients with pretransplant malignancies. Transplant Proc. (2010) 42:3006–10. 10.1016/j.transproceed.2010.08.012 20970594

[141] Oliveira GH Hardaway BW Kucheryavaya AY Stehlik J Edwards LB Taylor DO. Characteristics and survival of patients with chemotherapy- induced cardiomyopathy undergoing heart transplantation. J Heart Lung Transplant. (2012) 31:805–10. 10.1016/j.healun.2012.03.018 22551930

[142] DePasquale EC Nasir K Jacoby DL. Outcomes of adults with restric- tive cardiomyopathy after heart transplantation. J Heart Lung Transplant. (2012) 31:1269–1275. 10.1016/j.healun.2012.09.018 23079066

[143] Lenneman AJ Wang L Wigger M Frangoul H Harrell FE Silverstein C. Heart transplant survival outcomes for adriamycin-dilated cardiomyopathy. Am J Cardiol. (2013) 111:609–12. 10.1016/j.amjcard.2012.10.048 23195041 PMC3563750

[144] Oliveira GH Qattan MY Al-Kindi S Park SJ. Advanced heart failure therapies for patients with chemotherapy-induced cardiomyopathy. Circ Heart Fail. (2014) 7:1050–8. 10.1161/CIRCHEARTFAILURE.114.001292 25415958

[145] Araujo-Gutierrez R Ibarra-Cortez SH Estep JD Bhimaraj A Guha A Hussain I. Incidence and outcomes of cancer treatment-related cardiomyopathy among referrals for advanced heart failure. Cardiooncology. (2018) 4:3. 10.1186/s40959-018-0029-y 32154004 PMC7048122

[146] Desai A Noor A Joshi S Kim AS. Takotsubo cardiomyopathy in cancer patients. Cardiooncology. (2019) 5:7. 10.1186/s40959-019-0042-9 32154014 PMC7048040

[147] Curigliano G Cardinale D Dent S Criscitiello C Aseyev O Lenihan D. Cardiotoxicity of anticancer treatments: epidemiology, detection, and management. CA Cancer J Clin. (2016) 66:309–25. 10.3322/caac.21341 26919165

[148] Oliveira GH Dupont M Naftel D Myers SL Yuan Y Tang WH. Increased need for right ventricular support in patients with chemotherapy-induced cardiomyopathy undergoing mechanical circulatory support: outcomes from the INTERMACS registry (interagency registry for mechanically assisted circulatory support). J Am Coll Cardiol. (2014) 63:240–8. 10.1016/j.jacc.2013.09.040 24161324

[149] Montisci A Micheletto G Sibilio S Donatelli F Tespili M Banfi C. Impella 5.0 supported oncological surgery as bridge to LVAD. ESC Heart Fail. (2021) 8:167–70. 10.1002/ehf2.12758 33161652 PMC7835545

[150] Potapov EV Antonides C Crespo-Leiro MG Combes A Färber G Hannan MM. 2019 EACTS Expert Consensus on long-term mechanical circulatory support. Eur J Cardiothorac Surg. (2019) 56:230–70. 10.1093/ejcts/ezz098 31100109 PMC6640909

[151] Mehra MR Canter CE Hannan MM Semigran MJ Uber PA Baran DA. The 2016 International Society for Heart Lung Transplantation listing criteria for heart transplantation: a 10-year update. J Heart Lung Transplant. (2016) 35:1–23. 10.1016/j.healun.2015.10.023 26776864

